# Supplementary material for: Content-rich biological network constructed by mining PubMed abstracts
Source: BMC Bioinformatics. 2004 Oct 8;5:147. doi: 10.1186/1471-2105-5-147 (PMC528731; doi:10.1186/1471-2105-5-147)
Supplement: Additional File 2 — The original results of the above study (non-essential files are deleted to keep the file size under the limit set by BMC bioinformatics). [file 1471-2105-5-147-S2.bz2 › chilibotAdditionalFile2/dip10/392ID8700872E9878/html/FLT1_PGF.html]

 


 **FLT1** and **PGF** 
  
Found 101 abstracts in PubMed, retrieved 10.  
 

 What does Google say? 
 PDF only 
| .edu only 

---

**Interactive relationship** (e.g. stimulation, inhibition, etc)

**Neutral relationship**- PlGF  [ **PGF** ]  promotes recruitment of VEGFR1  [ **FLT1** ]  HSCs from a quiescent to a proliferative microenvironment within the BM, favoring differentiation, mobilization, and reconstitution of hematopoiesis.  Ref: 12799282 Ann N Y Acad Sci< MedlineTA>Ann N Y Acad Sci,
- Furthermore, the VEGF  **PGF**  heterodimer activated intramolecular VEGF receptor cross talk through formation of Flk1  **Flt1**  heterodimers.  Ref: 12796773 Nat Med< MedlineTA>Nat Med,
- We show that while a significant change in the formation of endothelial surface growth factor VEGFR1  [ **FLT1** ]  complexes is predicted in the presence of PlGF  [ **PGF** ] , the number of VEGFR2 containing signaling complexes is relatively unchanged.  Ref: 12714333 Am J Physiol Heart Circ Physiol< MedlineTA>Am J Physiol Heart Circ Physiol,
- Here we report that placental growth factor  [ **PGF** ]   **PGF** , also known as PlGF  [ **PGF** ]  regulates inter and intramolecular cross talk between the VEGF RTKs  **Flt1**  and Flk1.  Ref: 12796773 Nat Med< MedlineTA>Nat Med,
- Vessels of PlGF  [ **PGF** ]  implants were lined by vWF cells, which were mostly flt 1  [ **FLT1** ] .  Ref: 12673673 Int J Cancer, 2003
- Placental Growth Factor  [ **PGF** ]  PlGF  [ **PGF** ]  competes with Vascular Endothelial Growth Factor VEGF for binding to VEGF Receptor 1 VEGFR1  [ **FLT1** ] , but does not bind VEGFR2.  Ref: 12714333 Am J Physiol Heart Circ Physiol< MedlineTA>Am J Physiol Heart Circ Physiol, 2003
- Placental growth factor  [ **PGF** ]  PlGF  [ **PGF** ]  and vascular endothelial growth factor VEGF are involved in placental angiogenesis through interactions with the VEGFR 1  [ **FLT1** ]  and VEGFR  [ **FLT1** ]  2 receptors.  Ref: 12740423 J Physiol< MedlineTA>J Physiol, 2003
- In this review, we describe the molecular properties of PlGF  [ **PGF** ]  and VEGFR 1  [ **FLT1** ]  and how this translates into an important role for PlGF  [ **PGF** ]  in the angiogenic switch in pathological angiogenesis, by interacting with VEGFR 1  [ **FLT1** ]  and synergizing with VEGF.  Ref: 12871269 J Thromb Haemost, 2003
- They belong to the larger family which also includes VEGF, placenta growth factor  [ **PGF** ]  PlGF  [ **PGF** ]  and VEGF B, VEGF C and VEGF D are ligands for the endothelial cell specific tyrosine kinase receptors VEGFR  [ **FLT1** ]  2 and VEGFR  [ **FLT1** ]  3.  Ref: 12888864 Thromb Haemost, 2003
- Placental growth factor  [ **PGF** ]  1 PlGF  [ **PGF** ]  1, which exclusively binds VEGFR 1  [ **FLT1** ] , decreased hyperoxia induced retinal vaso obliteration from 22.2% to 5.1%.  Ref: 12840058 J Clin Invest, 2003
- Even though VEGF and  **PGF**  both bind  **Flt1** ,  **PGF**  uniquely stimulated the phosphorylation of specific  **Flt1**  tyrosine residues and the expression of distinct downstream target genes.  Ref: 12796773 Nat Med< MedlineTA>Nat Med, 2003

**Non-interactive relationship** (e.g. studied together, co-existance, homology, etc.)

- Role of PlGF  [ **PGF** ]  in the intra and intermolecular cross talk between the VEGF receptors  **Flt1**  and Flk1.  Ref: 12796773 Nat Med< MedlineTA>Nat Med, 2003
- and displacement of VEGF from VEGFR1  [ **FLT1** ]  to VEGFR2 by PlGF  [ **PGF** ] , causing increased signaling through VEGFR2.  Ref: 12714333 Am J Physiol Heart Circ Physiol< MedlineTA>Am J Physiol Heart Circ Physiol, 2003
- The mechanism whereby PlGF  [ **PGF** ]  enhanced early phases of BM recovery was mediated directly through rapid chemotaxis of readily available VEGFR1  [ **FLT1** ]  BM repopulating and progenitor cells.  Ref: 12799282 Ann N Y Acad Sci< MedlineTA>Ann N Y Acad Sci, 2003
- Plasma elevation of placental growth factor  [ **PGF** ]  PlGF  [ **PGF** ] , which signals through VEGFR1  [ **FLT1** ] , but not VEGFR2, restored hematopoiesis during the early and late phases following BM suppression.  Ref: 12799282 Ann N Y Acad Sci< MedlineTA>Ann N Y Acad Sci, 2003
- activation of VEGFR 1  [ **FLT1** ]  by PlGF  [ **PGF** ]  1 is a selective strategy for preventing oxygen induced retinal ischemia without provoking retinal neovascularization.  Ref: 12840058 J Clin Invest, 2003
- Placental growth factor  [ **PGF** ]  and its receptor, vascular endothelial growth factor receptor 1  [ **FLT1** ]  novel targets for stimulation of ischemic tissue revascularization and inhibition of angiogenic and inflammatory disorders.  Ref: 12871269 J Thromb Haemost, 2003
- Activation of  **Flt1**  by  **PGF**  resulted in intermolecular transphosphorylation of Flk1, thereby amplifying VEGF driven angiogenesis through Flk1.  Ref: 12796773 Nat Med< MedlineTA>Nat Med, 2003
- In contrast to VEGF and its receptor VEGFR  [ **FLT1** ]  2, PlGF  [ **PGF** ]  and its receptor VEGFR 1  [ **FLT1** ]  have been largely neglected and therefore their potential for therapy has not been previously explored.  Ref: 12871269 J Thromb Haemost, 2003
- Synergistic effects observed in that experiment thus appear not to be due to VEGF displacement, but to a shift from VEGF VEGFR1  [ **FLT1** ]  to PlGF  [ **PGF** ]  VEGFR1  [ **FLT1** ]  complexes and an increase in total VEGFR1  [ **FLT1** ]  complexes.  Ref: 12714333 Am J Physiol Heart Circ Physiol< MedlineTA>Am J Physiol Heart Circ Physiol, 2003
- Thus, these preclinical studies show proof of principle that PlGF  [ **PGF** ]  and VEGFR 1  [ **FLT1** ]  are promising therapeutic targets to treat angiogenesis and inflammation related disorders.  Ref: 12871269 J Thromb Haemost, 2003
- This synergy has been hypothesized to be due to a combination of signaling by PlGF  [ **PGF** ]  through VEGFR1  [ **FLT1** ] .  Ref: 12714333 Am J Physiol Heart Circ Physiol< MedlineTA>Am J Physiol Heart Circ Physiol, 2003
